# Effects of Loud Noise Exposure on DNA Integrity in Rat Adrenal Gland

**DOI:** 10.1289/ehp.7249

**Published:** 2004-09-22

**Authors:** Giada Frenzilli, Paola Lenzi, Vittoria Scarcelli, Francesco Fornai, Antonio Pellegrini, Paola Soldani, Antonio Paparelli, Marco Nigro

**Affiliations:** Dipartimento di Morfologia Umana e Biologia Applicata, University of Pisa, Pisa, Italy

**Keywords:** adrenal gland, comet assay, DNA damage, loud noise, rat

## Abstract

Loud noise is generally considered an environmental stressor causing negative effects on acoustic, cardiovascular, nervous, and endocrine systems. In this study, we investigated the effects of noise exposure on DNA integrity in rat adrenal gland evaluated by the comet assay. The exposure to loud noise (100 dBA) for 12 hr caused a significant increase of DNA damage in the adrenal gland. Genetic alterations did not decrease 24 hr after the cessation of the stimulus. We hypothesize that an imbalance of redox cell status is responsible for the induction and persistence of noise-induced cellular damage.

During daily life, people are exposed to potentially hazardous noise levels related to work environment, urban traffic, household appliances, discos, and the like (Kawecka-Jaszcz 1991; Lang et al. 1992). The World Health Organization (Berglund et al. 1999) estimated that approximately 20% of the European population is exposed to noise generated by urban traffic > 65 dBA, a level regarded as a maximum safety threshold, whereas 40% of Europeans are exposed to noise levels between 55 and 65 dBA, which might be responsible for several disorders of both auditory and extra-auditory organs (Berglund et al. 1999).

Extraauditory effects of noise have been related to psychophysiologic stress and the involvement of the pituitary–adrenocortical axis (Axelrod and Reisine 1984; Ising and Braun 2000). Most studies on the effects of noise exposure on the hypothalamus–pituitary–adrenocortical axis have been performed by measuring behavioral, endocrine, and biochemical variables (Alario et al. 1987; Armario et al. 1984; Borrell et al. 1980), whereas few studies have investigated the cellular effects induced by exposure to noise stress. Among these, Pellegrini et al. (1997) and Soldani et al. (1999) demonstrated the occurrence of ultrastructural modifications in the adrenal gland of noise exposed rats. Moreover, recent findings showed that ultra-structural alterations in the rat myocardium detected after loud noise exposure were also accompanied by DNA damage (Lenzi et al. 2003).

The purpose of the present study was to investigate whether levels of loud noise comparable with those present in modern daily life (Baker 1993; Berglund et al. 1999; Brüel 1970; Figure 1) were able to produce DNA damage in rat adrenal gland for the same doses and time intervals previously detected as effective for inducing cellular alterations in the heart (Lenzi et al. 2003).

## Materials and Methods

### Animals.

Male Wistar rats weighing 200–250 g (Harlan Labs, San Pietro al Natisone, Italy) were used for the experiments. Animals were housed in the animal facility, fed *ad libitum*, and kept under closely controlled environmental conditions (12 hr light:dark cycle, lights on between 0700 and 1900 hr; room temperature, 21°C). Animals were treated in accordance with the *Guidelines for the Care and Use of Laboratory Animals* (National Institutes of Health 1996). All possible efforts were made to reduce animal suffering and minimize the number of animals used.

### Experimental procedures.

Noise level was set at 100 dBA by the use of two loud speakers (15 W) (Lenzi et al. 2003) and lasted for 12 hr. Control rats were placed in the same kind of cage without being exposed to noise. Animals were randomly assigned to experimental and control groups, each consisting of four specimens. Experimental rats were sacrificed either soon after cessation of the noise stimulus or 24 hr later by decapitation, to avoid the interference of deep anesthesia with DNA integrity, and the adrenal gland was immediately dissected.

### Light microscopy.

To check for potential occurrence of cell death, we processed tissue samples using routine histologic procedures. Briefly, 8-μm-thick sections were cut with a microtome and stained with hematoxylin-eosin and toluidine blue. No cell death was observed.

### Evaluation of DNA damage.

**Figure 2:**
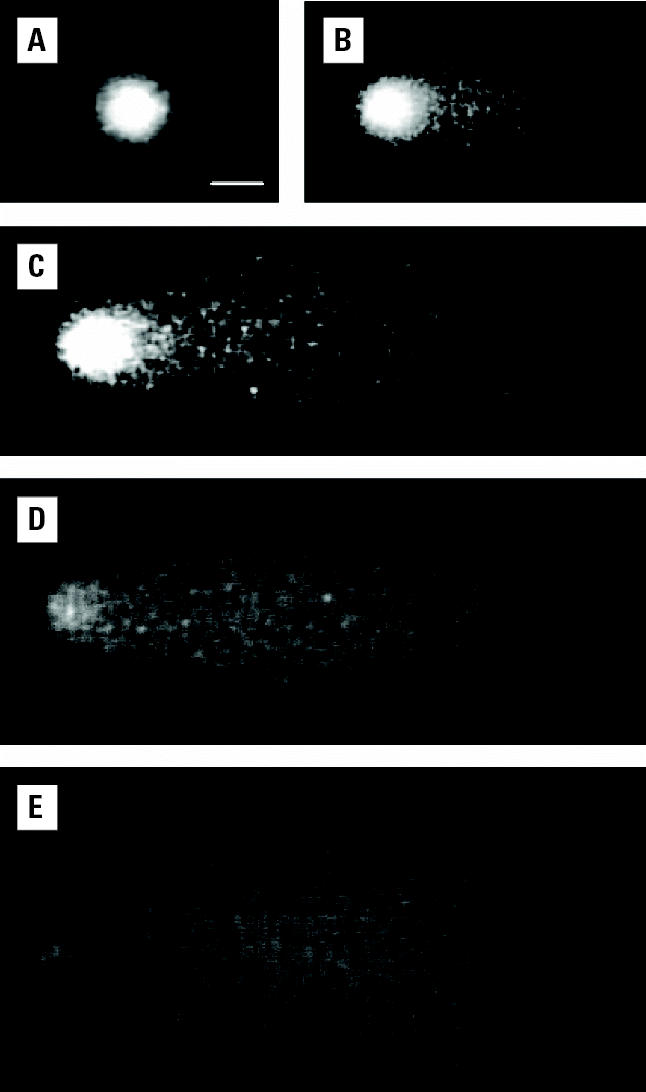
Images of ethidium-bromide–stained nuclei exhibiting different degrees of DNA damage after electrophoresis. The amount of DNA damage increases from *A* to *E*, as shown by the percentage of “tail” DNA: (*A*) 0.5%, (*B*) 10%, (*C*) 45%, (*D*) 93%, and (*E*) 99%. Bar = 20 μm.

We evaluated DNA integrity in rat adrenal gland by the use of alkaline single-cell gel electrophoresis or comet assay, according to Singh et al. (1988), with minor modifications (Lenzi et al. 2003). Electrophoretic DNA migration is proportional to the level of DNA damage producing cometlike images under a fluorescence microscope (magnification 200×) (Figure 2). We used an image analyzer (Komet, version 4; Kinetic Imaging Ltd., Bromborough, UK) to quantify the percentage of DNA migrated in the tail of at least 100 cells per animal. We used multifactor analysis of variance to assess the significance of factor effects such as animals, slides, and doses. For statistical analysis we used the software Statgraphics Plus for Windows (version 2.1; Microsoft Corp., Redmond, WA, USA).

## Results

**Figure 3:**
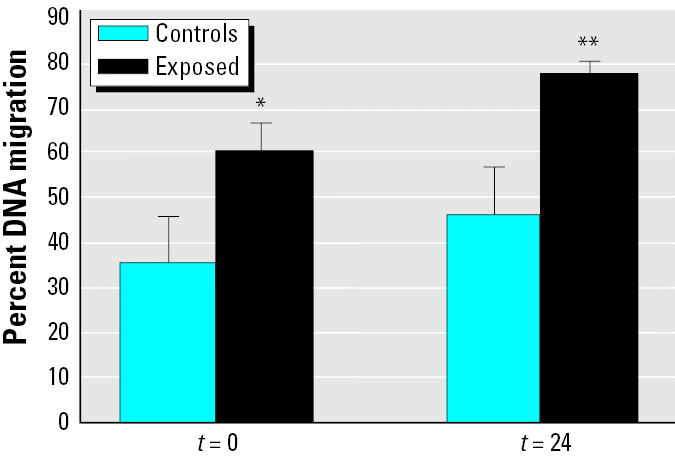
DNA damage induced by loud noise exposure in rat adrenal gland cells soon after 12 hr of noise exposure (*t* = 0) and 24 hr after the cessation of the stimulus (*t* = 24). Data are expressed as mean ± SD of DNA migrations.
*p < 0.01. **p < 0.001.

We evaluated the effect of loud noise on the presence of DNA damage in single cells dissociated from adrenal gland as the percentage of migrated DNA after electrophoresis in exposed and control rats. We observed a significant increase of DNA migration (*p* < 0.001), compared with controls, in the adrenal gland soon after the cessation of acoustic stress, as shown in Figure 3. This pattern of DNA migration persisted 24 hr after noise exposure, suggesting the absence of recovery (Figure 3). Light microscopy did not reveal the occurrence of cell death. This finding excludes the possibility that the number of strand breaks observed in the present study is due to nonspecific loss of DNA integrity related to cell death processes, providing supporting evidence of a genotoxic effect induced by loud noise.

## Discussion

This study demonstrates that loud noise exposure produces a significant loss of DNA integrity in the rat adrenal gland. This effect persisted almost unchanged 24 hr after the cessation of the stimulus. We can exclude the possibility that the elevation of DNA strand breaks was due to cell-death–associated fragmentation; indeed, light microscopy revealed a negligible occurrence of necrotic events. The same level and duration of the acoustic stress (100 dBA for 12 hr) were previously demonstrated to be effective in inducing ultrastructural alterations in rat adrenal cells, mainly involving the mitochondria and endoplasmic reticulum (Pellegrini et al. 1997). The adrenal gland is known to react to stressful stimuli, including noise. According to Ising and Braun (2000), habitual noise produces sympathetic activation and chronic increases in noradrenaline; nonhabitual noise produces an acute increase of noradrenaline and adrenaline; and extremely intense noise produces a defeat reaction with an increase of cortisol and adrenal stress hormone. The intense functional stimulation has been reported as potential cause for morphologic changes in subcellular structure, involving those organelles where steroids are synthesized, such as smooth endoplasmic reticulum and mitochondria (Simpson and Waterman 1988; Soldani et al. 1999).

Concerning the persistence of genetic damage, it is noteworthy that DNA single-strand breaks are usually repaired within 15 min and that DNA double-strand breaks are repaired within 2 hr (Plappert et al. 1997; Vijayalaxmi et al. 1993). Thus, such a maintenance of genotoxic effects 24 hr after noise exposure might be the consequence of a long-lasting clastogenic agent.

Our results on DNA damage might be interpreted as the output of two main events, namely, the clastogenic effect of oxyradicals and/or the DNA repair of oxidized bases, which implies the expression of alkali-labile sites, detected by the alkaline comet assay.

The negative effects of noise on cell structure and function were supposed to be, at least in part, mediated by the increase of reactive oxygen species (ROS) (Lenzi et al. 2003). ROS levels in the cochlea were found the be significantly higher 1 hr after exposure to 110 dB noise (Ohlemiller et al. 1999a), persisting after the cessation of the exposure (Ohlemiller et al. 1999b). In this respect, it is worthy to note that DNA is a main target of ROS toxicity (Cross et al. 1987; Lemasters et al. 1992). Oxidative damage of DNA is known to induce single-strand breaks and inter-/intrastrand cross-links (Caraceni et al. 1997). The involvement of ROS might play a causal role in the induction and persistence of genetic damage related to loud noise exposure also in extra-auditory organs. Indeed, Van Campen et al. (2002) reported an elevation of 8-hydroxy-2′-deoxyguanosine in brain and liver (besides the higher cochlear involvement) of rats exposed to loud noise (120 dB). According to these findings, the association between noise exposure, oxidative processes, and persisting DNA damage deserves further attention due to the long-lasting consequences in term of mutagenic and carcinogenic risk (Emerit 1994; Preston-Martin et al. 1989).

## Figures and Tables

**Figure 1:**
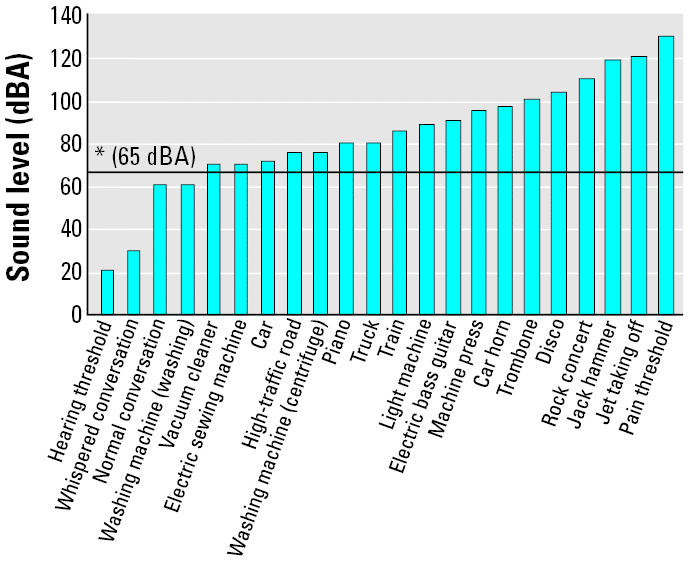
Sources and levels of noise exposure. Data represent a synthesis of data from different sources (see “Materials and Methods”).
*WHO safeness threshold limit (Berglund et al. 1999).

## References

[b1-ehp0112-001671] Alario P, Beato MJ, Trancho G (1987). Body weight gain, food intake and adrenal development in chronic noise stressed rats. Physiol Behav.

[b2-ehp0112-001671] Armario A, Castellanos JM, Balash J (1984). Adaptation of anterior pituitary hormones to chronic noise stress in rats. Behav Neural Biol.

[b3-ehp0112-001671] Axelrod J, Reisine TD (1984). Stress hormones: their interaction and regulation. Science.

[b4-ehp0112-001671] Baker DE 1993. Noise: The Invisible Hazard. Columbia, MO:University of Missouri Extension.

[b5-ehp0112-001671] Berglund B Lindvall T Schwela DH eds. 1999. Guidelines for Community Noise. London:World Health Organization.

[b6-ehp0112-001671] Borrell J, Torrellas A, Guaza C, Borrell S (1980). Sound stimulation and its effects on pituitary-adrenocortical function and brain catecholamines in rats. Neuroendocrinology.

[b7-ehp0112-001671] Brüel PV (1970). Do we measure damaging noise correctly?. B & K Technical Review.

[b8-ehp0112-001671] Caraceni P, De Maria N, Ryu HS, Colantoni A, Roberts L, Maidt ML (1997). Proteins but not nucleic acids are molecular targets for the free radical attack during reoxygenation of rat hepatocytes. Free Radic Biol Med.

[b9-ehp0112-001671] Cross DE, Halliwell B, Borish ET, Pryor WA, Ames BA, Saul RS (1987). Oxygen radicals and human disease. Ann Intern Med.

[b10-ehp0112-001671] Emerit I (1994). Reactive oxygen species, chromosome mutation, and cancer. Free Radical Biol Med.

[b11-ehp0112-001671] Kawecka-Jaszcz K (1991). Effect of professional work and environmental factors on arterial blood pressure. Med Pract.

[b12-ehp0112-001671] Ising H, Braun C (2000). Acute and chronic endocrine effects of noise: review of the research conducted at the Institute for Water, Soil and Air Hygiene. Noise Health.

[b13-ehp0112-001671] Lang T, Fouriaud C, Jacquinet-Salord MC (1992). Length of occupational noise exposure and blood pressure. Int Arch Occup Environ Health.

[b14-ehp0112-001671] Lemasters JJ Caldwell-Kenkel JC Gao W Nieminen AL Herman B Thurman RG 1992. Hypoxic, ischemic and reperfusion injury in the liver. In: Pathophysiology of Reperfusion Injury (Das DK, ed). Boca Raton, FL:CRC, 101–135.

[b15-ehp0112-001671] Lenzi P, Frenzilli G, Gesi M, Ferrucci M, Lazzeri G, Fornai F (2003). DNA damage associated with ultrastructural alterations in rat myocardium after loud noise exposure. Environ Health Perspect.

[b16-ehp0112-001671] National Institutes of Health 1996. Guidelines for Care and Use of Laboratory Animals. Washington, DC:National Academies Press.

[b17-ehp0112-001671] Ohlemiller KK, McFadden SL, Ding DL, Flood DG, Reaume AG, Hoffman EK (1999a). Targeted deletion of the cytosolic Cu/Zn-superoxide dismutase gene (Sod1) increases susceptibility to noise-induced hearing loss. Audiol Neurootol.

[b18-ehp0112-001671] Ohlemiller KK, Wright JS, Dugan LL (1999b). Early elevation of cochlear reactive oxygen species following noise exposure. Audiol Neurootol.

[b19-ehp0112-001671] Pellegrini A, Soldani P, Gesi M, Lenzi P, Natale G, Paparelli A (1997). Effect of varying noise stress duration on rat adrenal gland: an ultrastructural study. Tissue Cell.

[b20-ehp0112-001671] Plappert UG, Stocker B, Fender H, Fliedner TM (1997). Changes in the repair of blood cells as a biomarker for chronic low-dose exposure to ionizing radiation. Environ Mol Mutagen.

[b21-ehp0112-001671] Preston-Martin S, Thomas DC, Wright WE, Henderson BE (1989). Noise trauma in the aetiology of acoustic neuromas in men in Los Angeles County. 1978–1985. Br J Cancer.

[b22-ehp0112-001671] Simpson ER, Waterman MR (1988). Regulation of the synthesis of steroidogenic enzymes in adrenal cortical cells by ACTH. Annu Rev Physiol.

[b23-ehp0112-001671] Singh NP, McCoy MT, Tice RR, Schneider EL (1988). A simple technique for quantitation of low levels of DNA damage in individual cells. Exp Cell Res.

[b24-ehp0112-001671] Soldani P, Gesi M, Lenzi P, Natale G, Fornai F, Pellegrini A (1999). Long-term exposure to noise modifies rat adrenal cortex ultrastructure and corticosterone plasma levels. J Submicrosc Cytol Pathos.

[b25-ehp0112-001671] Van Campen LE, Murphy WJ, Franks JR, Mathias PI, Toraason MA (2002). Oxidative DNA damage is associated with intense noise exposure in the rat. Hear Res.

[b26-ehp0112-001671] Vijayalaxmi, Strauss GHS, Tice RR (1993). An analysis of γ -ray-induced DNA damage in human blood leukocytes, lymphocytes and granulocytes. Mutat Res.

